# Lateral canal peripheral positional vertigo diagnosis: a neurophysiological approach

**DOI:** 10.3389/fneur.2025.1643458

**Published:** 2025-07-25

**Authors:** Francisco Carlos Zuma E Maia, Bernardo Faria Ramos, Melissa Castillo-Bustamante, Renato Valerio Cal, Jorge Madrigal

**Affiliations:** ^1^Clinica Maia, Canoas, Brazil; ^2^Department of Otorhinolaryngology, Health Sciences Center, Federal University of Espírito Santo, Vitoria, Brazil; ^3^Department of Otorhinolaryngology, Clinica Universitaria Bolivariana, Universidad Pontificia Bolivariana, Medellín, Colombia; ^4^Department of Otorhinolaryngology, University Center of the State of Pará, Belem, Brazil; ^5^Centro de Vértigo y Mareo, Mexico City, Mexico

**Keywords:** benign paroxysmal positional vertigo, lateral canal BPPV, horizontal canal BPPV, apogeotropic nystagmus, geotropic nystagmus

## Abstract

Lateral semicircular canal peripheral positional vertigo (LC-PPV) is a common condition, even though accurate determination of the affected side remains a diagnostic challenge that is crucial for effective treatment. The aim of this study is to provide a systematic diagnostic approach for lateralization in LC-PPV, based on analysis of endolymphatic flow dynamics elicited by the Bow and Lean test and supine roll test.

## Introduction

The sensor for angular head acceleration in the yaw plane is the lateral semicircular canal (LC), which is inclined approximately 30 degrees relative to the horizontal plane. According to Ewald’s second law, ampullopetal endolymphatic flow (toward the ampulla) is excitatory for this canal, whereas ampullofugal flow (away from the ampulla) produces an inhibitory stimulus.

The LC is the second most frequently affected semicircular canal in peripheral positional vertigo (PPV) (1). This variant is characterized by horizontal nystagmus that beats either toward the ground (geotropic) or toward the ceiling (apogeotropic) during positional testing. The initial location of otoliths may be free-floating in the non-ampullary arm of the LC (geotropic variant) or in the ampullary arm or attached to the cupula (apogeotropic variant) (2).

The primary diagnostic test for LC-PPV is the Supine Roll Test (SRT), in which the head is rotated 90 degrees to each side while the patient is on supine position. The direction and intensity of the induced nystagmus are critical for identifying the affected side. In this test, nystagmus typically beats toward the undermost ear in the geotropic variant and toward the uppermost ear in the apogeotropic variant. Additionally, nystagmus is more intense when the head is turned toward the affected side in geotropic LC-PPV and toward the unaffected side in the apogeotropic variant. However, distinguishing subtle differences in nystagmus intensity during the SRT can sometimes be challenging, possibly due to variations in otolith volume or distribution within the canal (3, 4).

**Figure 1:**
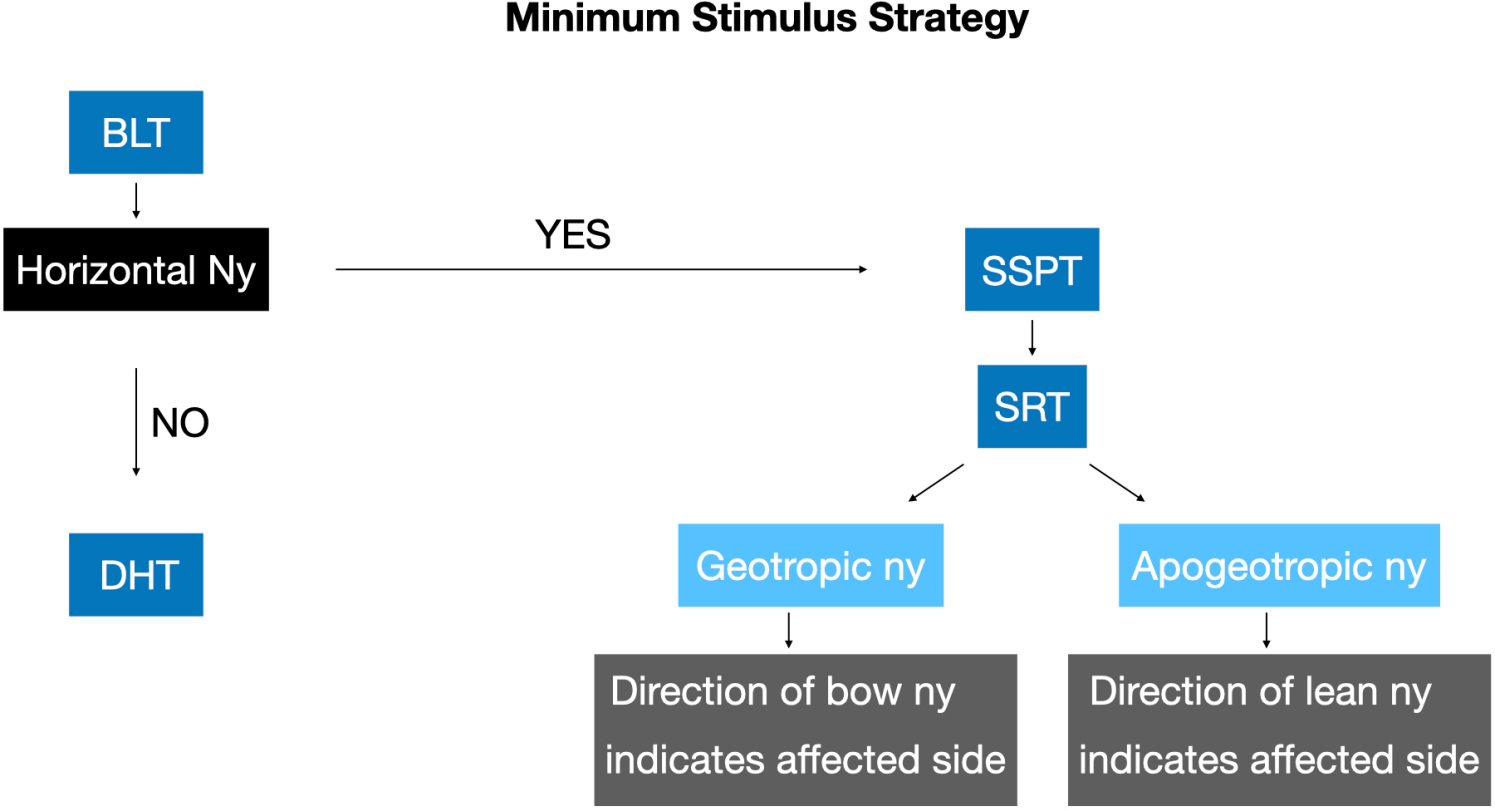
Diagnostic algorithm based on variant identification (geotropic vs. apogeotropic) combined with Bow and Lean test findings. BLT, Bow and Lean test; DHT, Dix Hallpike test; Ny, nystagmus; SRT, supine roll test; SSPT, seated supine positioning test.

The Minimum Stimulus Strategy, first described by Asprella-Libonatti (5), is a stepwise approach based on nystagmus patterns elicited during a sequence of positional tests. This method aims to achieve successful treatment outcomes in the first session while minimizing vertigo symptoms. The Bow and Lean Test is performed with the patient in the seated position, with the head flexed forward and extended backward (6). The direction and intensity of nystagmus observed during this test may serve as key indicators for determining the affected side (7). If there is a horizontal nystagmus during the Bow and Lean Test, we may proceed with the Seated Supine Positioning Test (SSPT) succeeded by the SRT, given the clinical suspicion of LC-PPV. On the other hand, we may continue with the Dix Hallpike test (8) (Figure 1).

The objective of this study is to provide practical guidance for diagnosing and identifying the affected side in LC-PPV, based on an analysis of endolymphatic flow dynamics induced by the Bow and Lean Test and the SRT.

## Case descriptions

### Case 01

Male, 70 years old. Bedside examination at the ENT office with a video-Frenzel-goggle did not exhibited a spontaneous nystagmus with or without visual fixation. Bow test with a left beating nystagmus, Lean test with a right beat nystagmus and SRT with an apogeotropic nystagmus. We were unable to identify the difference in intensity of the nystagmus in the SRT. Therefore, we can assume that the otoliths were located in the ampullary arm of the right LC, because there was an apogeotropic nystagmus in the SRT (ampullary arm) and a right beating nystagmus in the lean test (Right LC) (Supplementary Video 1).

### Case 02

Female, 74 years old. Bedside examination at the ENT office with a video-Frenzel-goggle did not exhibited a spontaneous nystagmus with or without visual fixation. Bow test with a right beating nystagmus, Lean test with a left beat nystagmus and SRT with a geotropic nystagmus. We were unable to identify the difference in intensity of the nystagmus in the SRT. Therefore, we can assume that the otoliths were located in the non-ampullary arm of the right LC, because there was a geotropic nystagmus in the SRT (non-ampullary arm) and a right beating nystagmus in the bow test (Right LC) (Supplementary Video 2).

### Diagnosis assessment

The diagnosis and determination of the affected side in LC-PPV requires a structured sequence of positional testing. The evaluation begins with the Bow and Lean test performed by sequentially flexing and extending the patient’s head, while seated.

**Figure 2:**
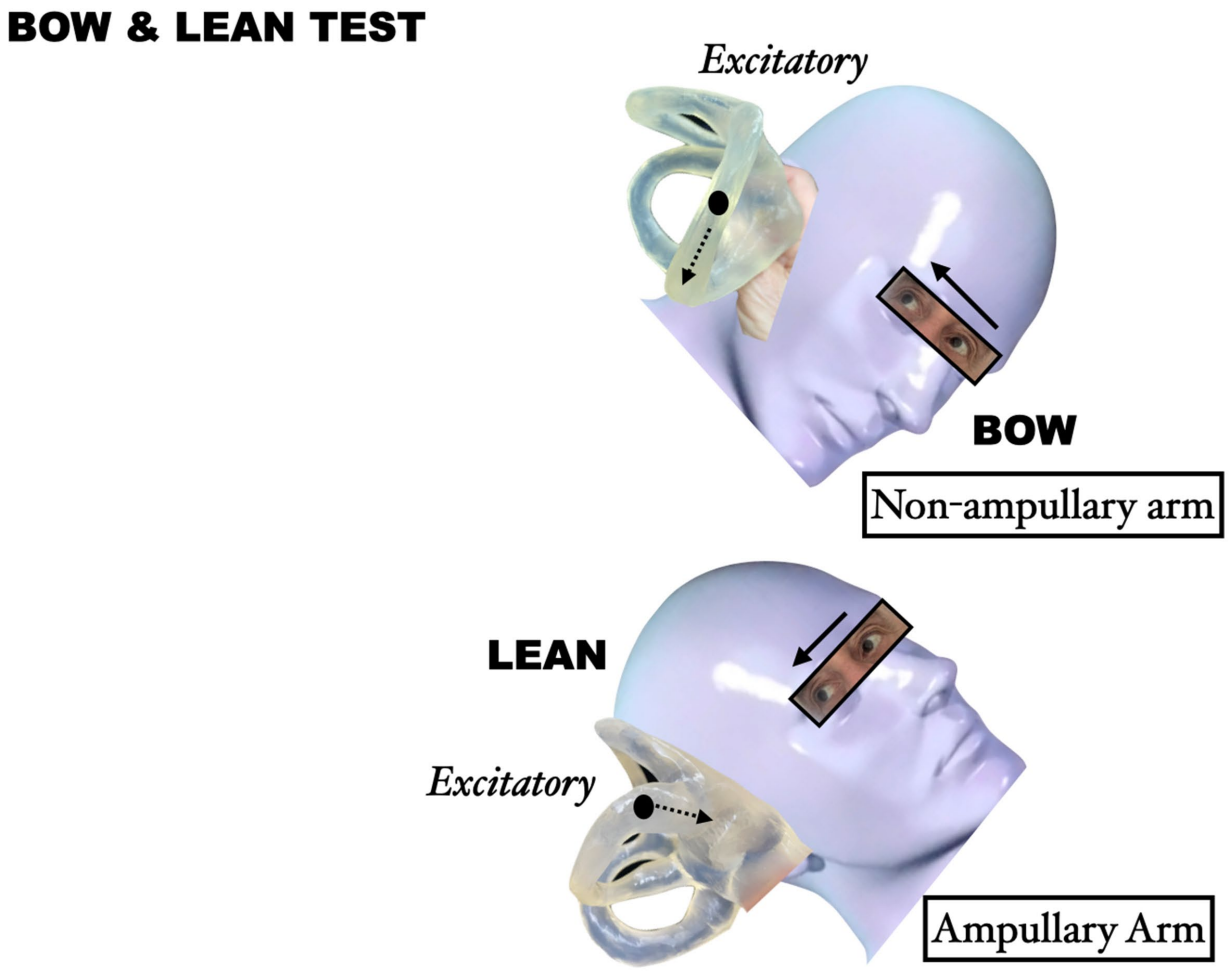
Endolymphatic flow during the Bow and Lean test.

In the geotropic variant (otoliths located in the non-ampullary arm of the LC), the Bow test induces ampullopetal movement of otolithic debris under the gravitational force, generating excitatory endolymphatic flow and consequent nystagmus beating toward the affected side. Conversely, in the apogeotropic variant, the Lean test produces ampullopetal movement, resulting in excitatory flow and nystagmus directed toward the affected side (9, 10) (Figure 2).

The evaluation continues with the Seated Supine Positioning Test (SSPT), which represents the natural transition between seated and supine positions required for SRT execution. While SSPT alone cannot differentiate between both variants, it provides valuable lateralization clues through characteristic nystagmus patterns, as it elicits nystagmus beating toward the unaffected side in geotropic variant and toward the affected side in apogeotropic variant. Importantly, since patients must be moved to supine position for SRT regardless, SSPT adds negligible additional stimulation while yielding useful diagnostic data.

The diagnostic sequence culminates with the SRT, where 90° head rotations produce direction-changing nystagmus: geotropic variants demonstrate nystagmus beating toward the undermost ear, while apogeotropic variants show nystagmus beating toward the uppermost ear.

Given the occasional difficulty in discerning nystagmus intensity differences during SRT (6, 7), we propose a diagnostic algorithm based on variant identification (geotropic vs. apogeotropic) combined with Bow and Lean test findings. In geotropic LC-PPV, the affected side corresponds to the direction of Bow test-evoked nystagmus. Conversely, in apogeotropic variant, the affected side matches the direction of Lean test-induced nystagmus (7). For instance, right geotropic LC-PPV (non-ampullary arm) manifests as right-beating nystagmus during the Bow test (excitatory stimulus) and geotropic nystagmus in SRT, as demonstrated in Case 02.

## Discussion

While less frequently encountered than posterior canal PPV, LC-PPV presents unique diagnostic and therapeutic challenges. Accurate identification of the affected side is crucial for successful treatment outcomes (9, 10). Although the supine roll test (SRT) remains the primary diagnostic tool for LC-PPV, reliance solely on nystagmus intensity differences during SRT may prove diagnostically challenging in certain cases (6, 7).

Complementary diagnostic tools, including pseudo-spontaneous nystagmus evaluation, Bow and Lean test, Seated Supine positioning test (SSPT), and walk-rotate-walk test, have been proposed as valuable adjuncts for lateralization (5, 6, 10, 11). Notably, clinical studies demonstrate that bowing and/or leaning-induced nystagmus can be observed in 70–90% of LC-PPV cases, providing critical diagnostic information when SRT findings are not clear (6, 12).

Based on our understanding of endolymphatic flow dynamics during positional testing, we propose a systematic diagnostic approach incorporating the following principles:

- The Bow and Lean test represents a minimally invasive, easily performed diagnostic test.
- Excitatory stimuli consistently produce nystagmus beating toward the affected side.
- The Bow test elicits an excitatory stimulus in geotropic LC-PPV (7).
- The Lean test generates an excitatory stimulus in apogeotropic LC-PPV (7).

These physiological principles support a diagnostic algorithm where SRT determined variant classification (geotropic or apogeotropic) combined with Bow and Lean test nystagmus direction provides reliable information to identify the affected side in LC-PPV. While many LC-PPV cases can be accurately diagnosed through SRT evaluation alone, Bow and Lean test findings serve as valuable confirmatory evidence, enhancing diagnostic certainty.

## Conclusion

The Minimum Stimulus Strategy facilitates the application of neurophysiological principles for diagnosing LC-PPV, as it provides multiple concordant data points for lateralization, while maintains patient comfort by minimizing provocative positional tests. Our findings demonstrate that combining variant identification through the Supine Roll Test (geotropic or apogeotropic) with nystagmus direction analysis during the Bow and Lean Test provides a reliable method for determining the affected side in LC-PPV cases, particularly when SRT nystagmus intensity differences are ambiguous.

## Supplementary material

SUPPLEMENTARY VIDEO 1 Right apogeotropic LC-PPV.

SUPPLEMENTARY VIDEO 2 Right geotropic LC-PPV.
